# Supplementary material for: Arabidopsis histone H3 lysine 9 methyltransferases KYP/SUVH5/6 are involved in leaf development by interacting with AS1-AS2 to repress KNAT1 and KNAT2
Source: Commun Biol. 2023 Feb 24;6:219. doi: 10.1038/s42003-023-04607-6 (PMC9958104; doi:10.1038/s42003-023-04607-6)

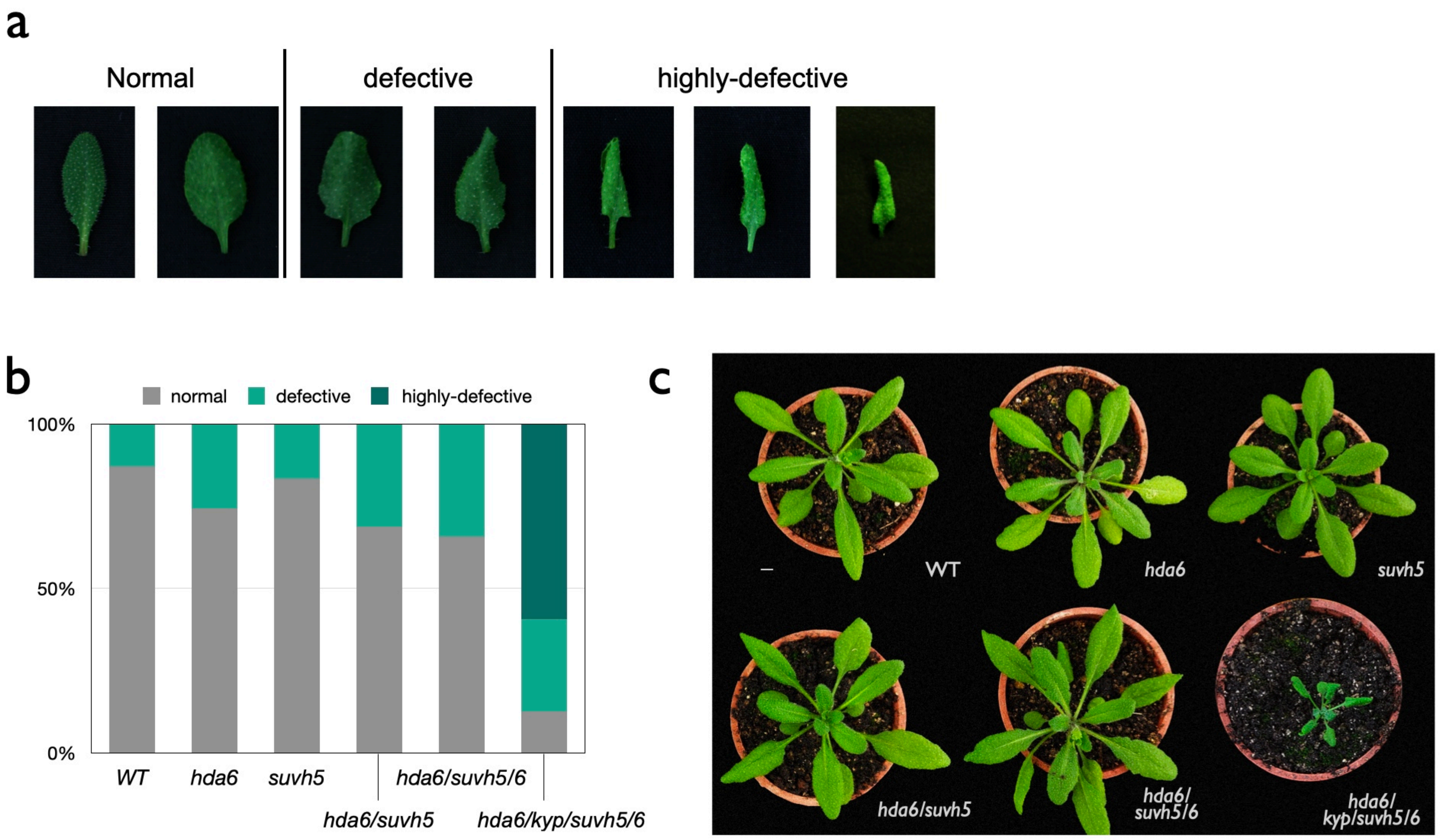

Figure S1. *Arabidopsis* KYP, SUVH5 and SUVH6 are involved in leave development.

(a) Sample photos of the normal, defected and highly-defected leaves.

(b, c) Quantitative analysis (b) and phenotypes (c) of lesves on WT, *hda6, suvh5, hda6/shvu5, hda6/suvh5/6* and *hda6/kyp/suvh5/6* mutant plants. The fourth pair rosette leaves of each plant were classified as normal, defected and highly-defected leaves. Frequency is defined as the ratio of the number of leaves examined. Bars= 5mm.

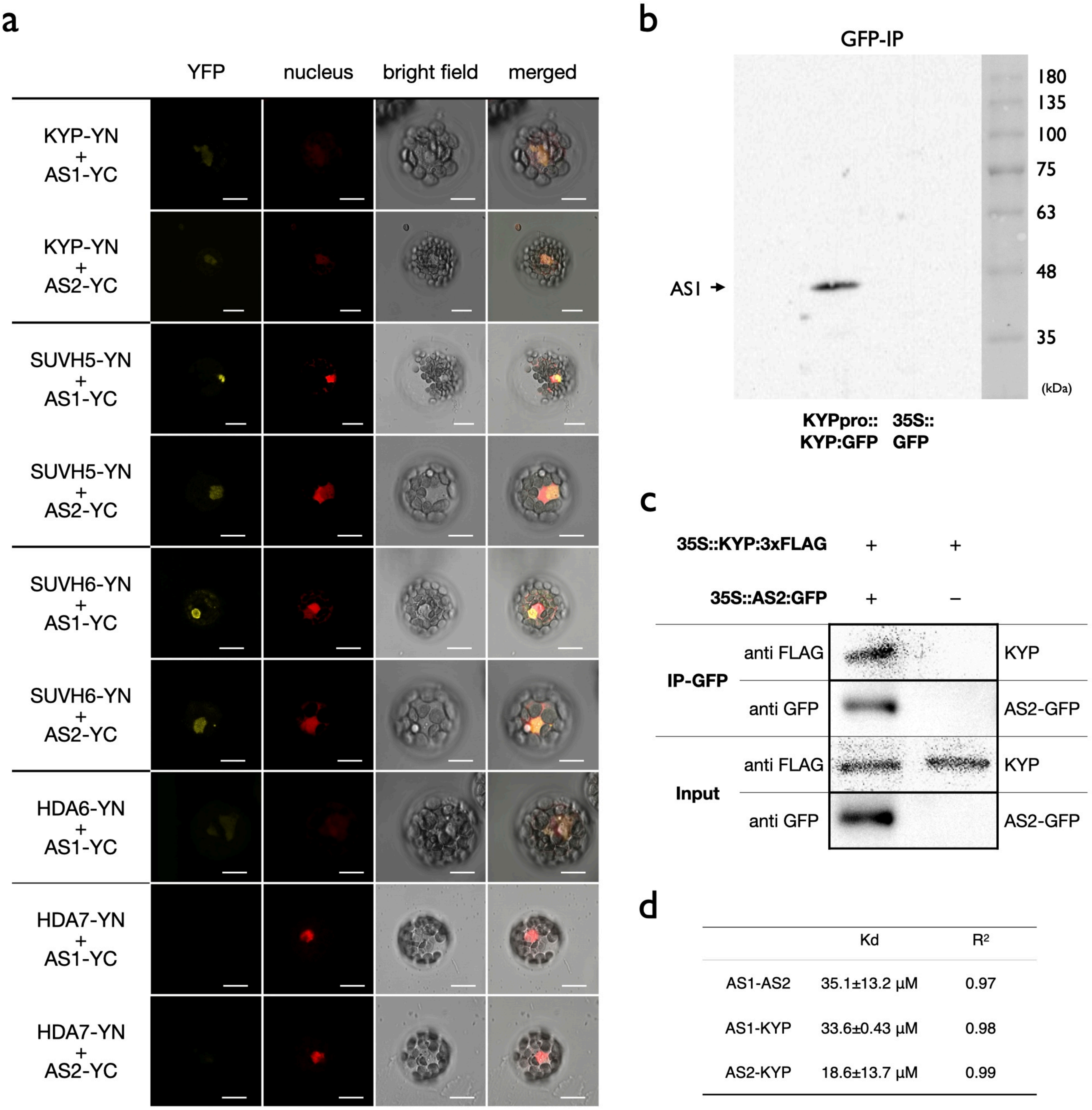

	Kd	R^2
AS1-AS2	35.1±13.2 μM	0.97
AS1-KYP	33.6±0.43 μM	0.98
AS2-KYP	18.6±13.7 μM	0.99

Figure S2. KYP, SUVH5 and SUVH6 interact with AS1 and AS2.
(a) BiFC assays in *Arabidopsis* protoplast showing interaction of KYP, SUVH5 and SUVH6 with AS1 and AS2 in living cells. Different regions of KYP, SUVH5, SUVH6 and AS1/2 proteins were fused with the N terminus (YN) or C terminus (YC) of YFP and co-delivered into protoplasts. The nucleus was indicated by mCherry carrying a nuclear localization signal. Bars= 10μm.
(b) Western blot showing AS1 co-immunoprecipitated by KYP:GFP in transformed *Arabidopsis*.
(c) Co-IP of KYP:3xFLAG and AS2:GFP in transformed *Arabidopsis* protoplasts. Western blot was performed with the indicated antibodies.
(d) Table of the dissociation rate constant (Kd) values were measured among AS1, AS2, and KYP recombinant protein pairs in the QCM assay. The average Kd and standard deviation values were obtained from 3 replicates of AS1-AS2, AS1-KYP, and AS2-KYP pairs.

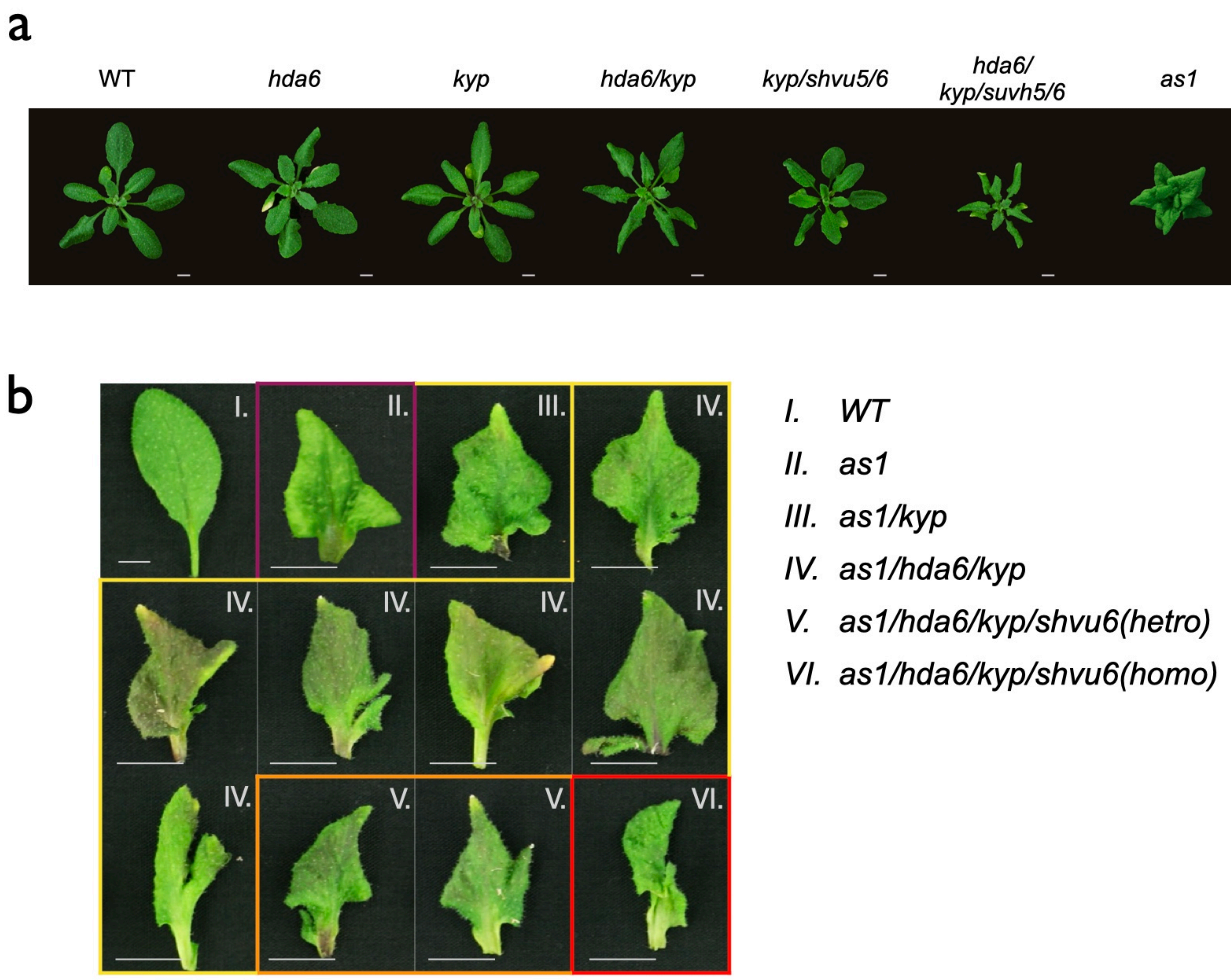

Figure S3. the function of KYP/SUVH5/6 in leaf development is depending on AS1.
(a) Leaf development phenotype of WT, *hda6, kyp, kyp/hda6, kyp/suvh5/6* and *hda6/kyp/suvh5/6* mutant plants.
(b) Leaf development phenotype of WT and indicated mutant plants.
Bars= 5mm. Plants were grown at 22°C under long-day for 20 days.

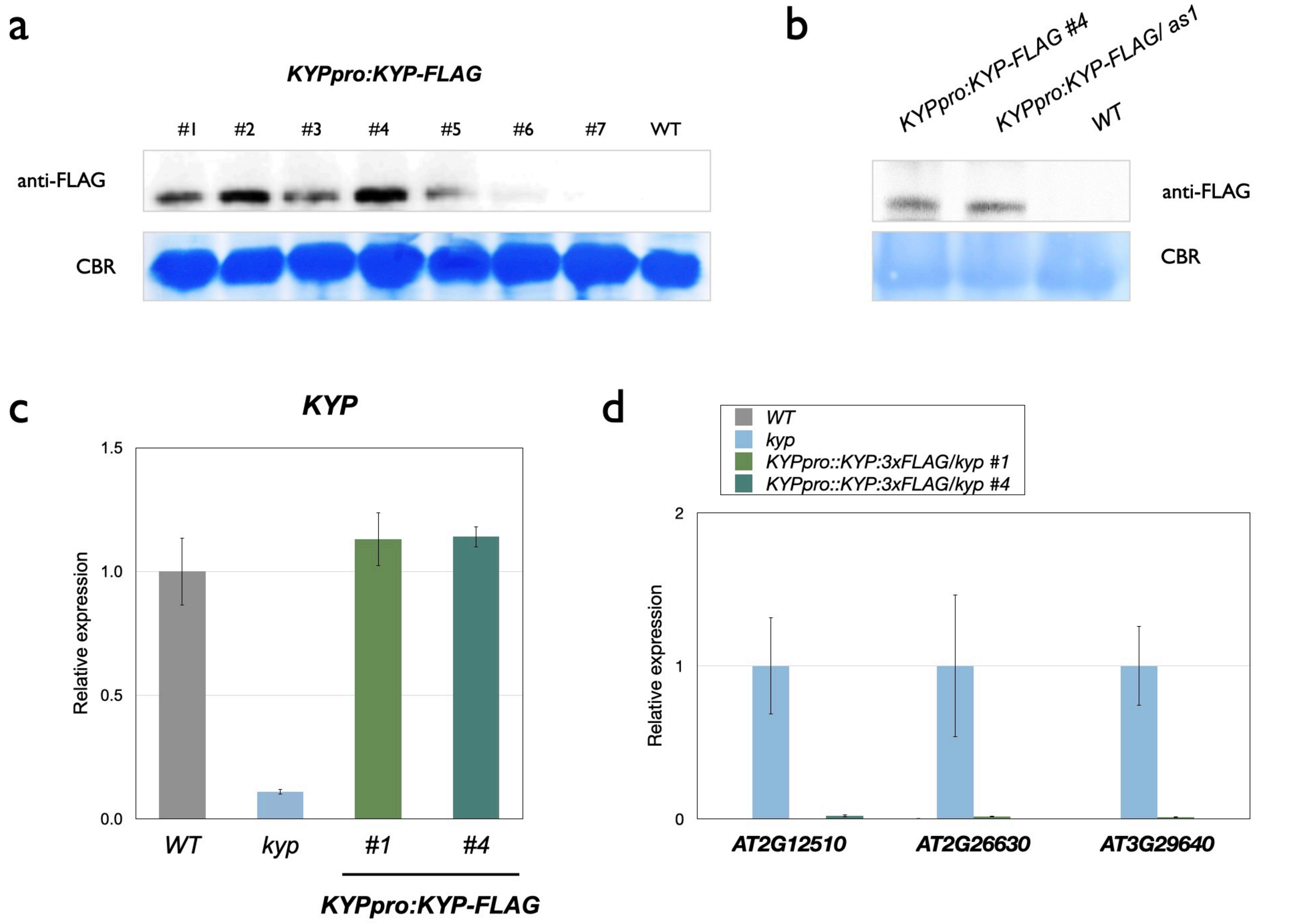

Figure S4. The transcription and translation analysis of *KYPpro:KYP-FLAG* transgenic lines.
(a, b) Total proteins were extracted from the *KYPpro:KYP-FLAG* transgenic lines, followed by immunoblot using an anti-FLAG antibody. CBR indicated Coomassie Brilliant Blue Staining loading control.
(c, d) Expression of *KYP* and indicated TEs was analyzed by qRT-PCR. RNA was extracted from 10-day-old plants grown under LD conditions. *UBQ10* was used as an internal control. The experiments were repeated three times with similar results. Error bars indicate ±SD.

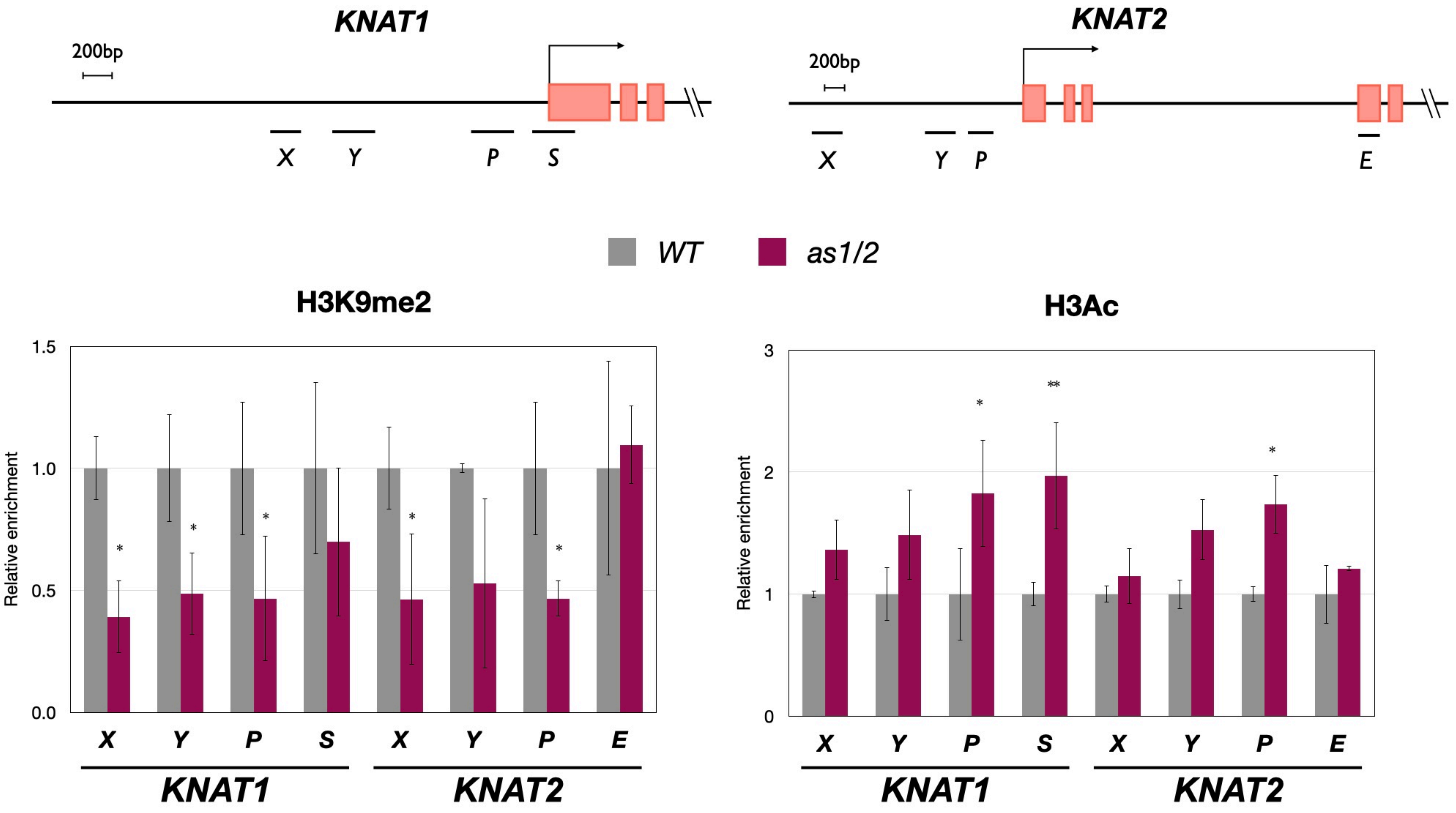

Figure S5. The H3K9me2 and H3Ac of *KNAT1* and *KNAT2* in *as1/2*.
ChIP analysis of H3K9me2 and H3Ac level on *KNAT1* and *KNAT2*. Plants were grown under LD for 10 days. The amounts of DNA after ChIP were quantified by qPCR and normalized to *TA3* or *TUB2*. Data points represent average of three technical replicates. Error bars correspond to standard deviations from three biological replicates. *P< 0.05, **P< 0.005 (Student's t-test).

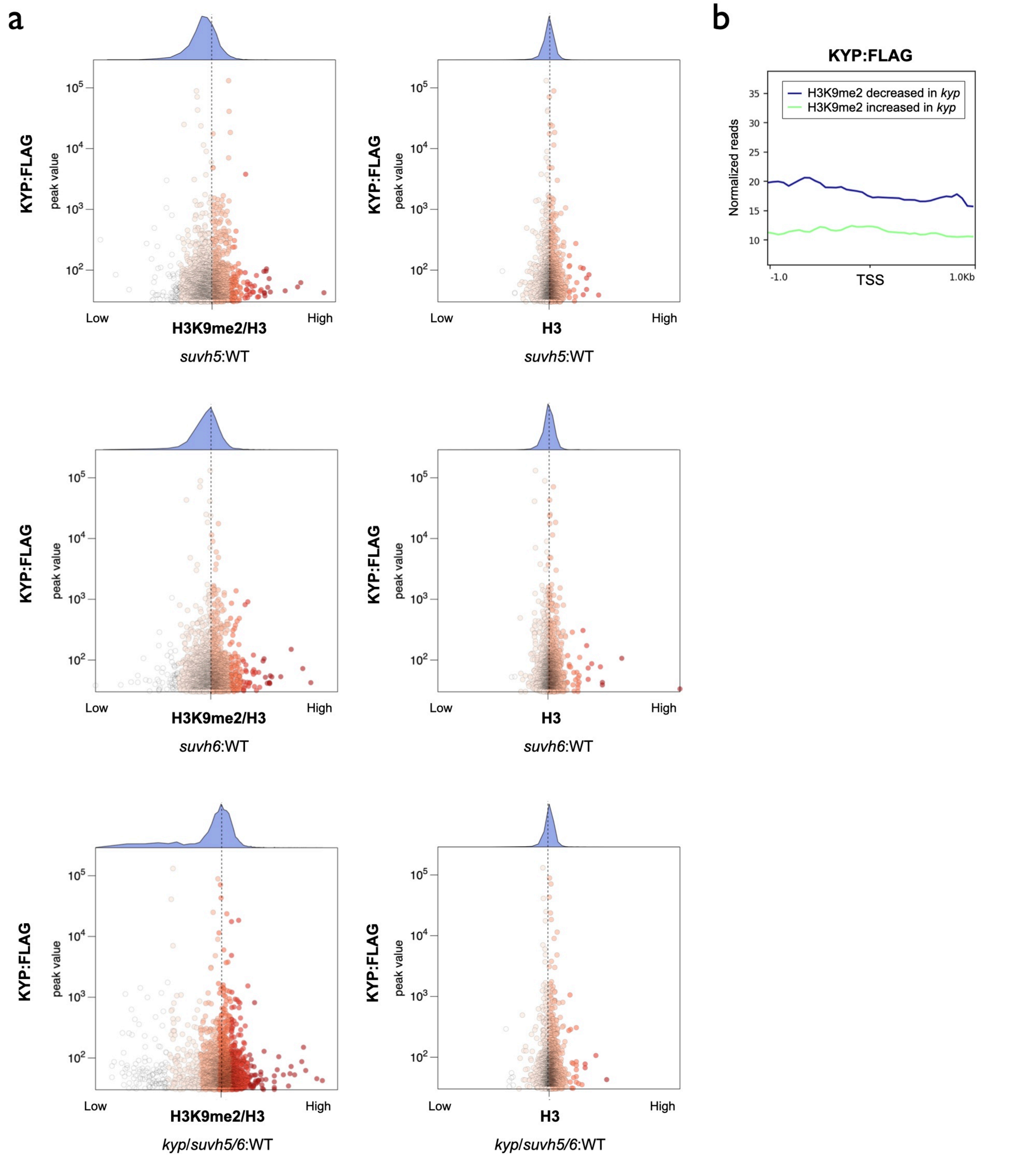

Figure S6. The binding of KYP is correlated with H3K9me2 level change in *kyp/suvh5/6.*

(a) X-Y scatter plots showing the relative enrichment of H3K9me2 level in *suvh5*: WT, *suvh6*: WT and *kyp*/*suvh5/6*: WT with the binding level of KYP. The value of H3K9me2/H3 indicated relative H3K9me2 level of *kyp* compared to WT and normalized to H3. The dotted line indicated average value.

(b) The mean density of KYP enrichment in the gene groups which showing decreased- or increased- H3K9me2 level in *kyp*. The average KYP enrichment signal within 1 kb genomic regions from up-stream and down-stream of TSS were shown. The KYP enrichment level were marked in blue (H3K9me2 decreased genes in *kyp*) or green (H3K9me2 increased genes in *kyp*).

Table S1 Primers used in this study

Name	sequence	note
KNAT1 RTF	GGGAAGAGATGACAATATGGG	RT-PCR
KNAT1 RTR	TATGGACCGAGACGATAAGG	RT-PCR
KNAT2 RTF	TCATCTGACGAGGAACTGAG	RT-PCR
KNAT2 RTR	CGTCCATCATATCAAACGGC	RT-PCR
STM RTF	ACAGTGGTTACTTAGGGAGC	RT-PCR
STM RTR	TTGGGAAAGGATTGCCCAAG	RT-PCR
KNAT3 RTF	CATTTCCAAGAAGCACCGCC	RT-PCR
KNAT3 RTR	CTTGCGGTTGTGGTGTTAGC	RT-PCR
KNAT5 RTF	CTACCACCCTTACCACTGCG	RT-PCR
KNAT5 RTR	TATCGTTTCACCATCGGCGT	RT-PCR
KNAT6 RTF	ATCTgACgAggAACTgAgTg	RT-PCR
KNAT6 RTR	CTCTgATggCTTCCAATgAC	RT-PCR
NUC1 RTF	GACGTGATTGCTGCTGTCCA	RT-PCR
NUC1 RTR	GCCTTCTTGGCTGGGACTTT	RT-PCR
PAP15 RTF	TCTTCTGCTTTCTCTCGCCG	RT-PCR
PAP15 RTR	TTGACGGAATAGAGTGGGCG	RT-PCR
GRF4 RTF	CTATCCCAAAAGCAACCAACAC	RT-PCR
GRF4 RTR	GCAGCAGAGCCTTGTTCTTC	RT-PCR
CDKC2 RTF	GAGGAGCCTCCACCGATTTG	RT-PCR
CDKC2 RTR	GCGGTTATCGGAAACCCTTCT	RT-PCR
MLP328 RTF	AAGGAAGAGAGATGGCGACG	RT-PCR/ChIP
MLP328 RTR	CCTCCACCGCTTGTAGTGTT	RT-PCR/ChIP
ERF96 RTF	GAACGTGTGTGGCTTGGAAC	RT-PCR
ERF96 RTR	ATTGCTGCTTGGCCTCTCAT	RT-PCR
AT2G12510 RTF	CGTCCCATCAACCTACCACC	RT-PCR
AT2G12510 RTR	CGTACACGACTGCTATCCCC	RT-PCR
AT2G14190 RTF	TTCCAAGTCCAAGAGGGGGA	RT-PCR/ChIP
AT2G14190 RTR	GGTTGGTTCTGTTGCTGCTG	RT-PCR/ChIP
AT3G29640 RTF	TTGTTCTCGTCAGGGCATGG	RT-PCR
AT3G29640 RTR	TGACCTTTGACCACCTTTGC	RT-PCR
UBQ10 RTF	GATCTTTGCCGGAAAACAATTGGAGGAT[illegible]	RT-PCR
UBQ10 RTR	CGACTTGTCATTAGAAAGAAAGAGATAA[illegible]	RT-PCR
KNAT1 X_F	TACACGAACACAGATGAT	ChIP
KNAT1 X_R	CAGTGGAAGTGAGAGTAGG	ChIP
KNAT1 Y2_F	ACTGAAACAGAAAATAAGAGGAGGT	ChIP
KNAT1 Y2_R	TTGCATTCGAAATGTTTTCTTTTCC	ChIP
KNAT1 P_F	AACCATAGCCTGAAGTAGCC	ChIP
KNAT1 P_R	AAGACGTCGTTTGCTTTGGG	ChIP
KNAT1 S_F	CTCTTCATCTTACACCCATCC	ChIP
KNAT1 S_R	CCAGGACCATAATTGCTAC	ChIP
KNAT2 X2_F	CGTCGTCAAATGCGCCATAC	ChIP
KNAT2 X2_R	TAAGGGCAAGGGAATTGGGC	ChIP
KNAT2 Y_F	CTGTCGTTTTTATAAGGTTTG	ChIP
KNAT2 Y_R	CACTTATCGCACTTCTTGTTA	ChIP
KNAT2 P_F	AACCGATCCGGTTAGACAAC	ChIP
KNAT2 P_R	CTGTACGATTACATGGTTACG	ChIP
KNAT2 E_F	CAAAGAAGCAATGACCGCGA	ChIP
KNAT2 E_R	ACACATTCGAGAGGCGGATATT	ChIP
KNAT3_P1F	TGGATGGATGCAGTGTGGAC	ChIP
KNAT3_P1R	GAAGAAAACCGCAACGGACC	ChIP
KNAT5_P1F	CTCTCCCGTCACATTCTGGT	ChIP
KNAT5_P1R	GGAGGGAGGGAGGGAGTAAA	ChIP
NUC1 P1F	GCGCTACTTCTCCGCTTTGA	ChIP
NUC1 P1R	TTGTGTCAAGTGGCAACGGT	ChIP
PAP15 P1F	CTTTTTGACTCTTCCTATTCAGTCA	ChIP
PAP15 P1R	ACACTCATTTGTCGATCTTTAACG	ChIP
GRF4 P1F	GTGCCCTCGTCGGAAACTAA	ChIP
GRF4 P1R	GGCCCAAACTCTCCACCTAC	ChIP
CDKC2 P1F	TCGGTTGAATAATGGCGGCT	ChIP
CDKC2 P1R	ACCCGTAAGTTCCTTCACCA	ChIP
ERF96 P1F	ACTCACTCTCACACACACACTC	ChIP
ERF96 P1R	ACAGAATGCCGTTTCTCATGC	ChIP
AT2G14190 P1F	GAGAAGGCAGCGGAGGATTC	ChIP
AT2G14190 P1R	CGACGTGAAGCAAATCTGTCA	ChIP
AT3G29640 P1F	GAGCCGGACTTTTGATCCGA	ChIP
AT3G29640 P1R	TCATGCCCAACAACTGCTCA	ChIP
TA3-F	CTGCGTGGAAGTCTGTCAAA	ChIP
TA3-R	CTATGCCACAGGGCAGTTTT	ChIP
Actin RT1	AATGGAAGCTCCTGGAATCC	ChIP
Actin RT2	ATCGATGGACCTGACTCATC	ChIP
TUB2-F	ACAAACACAGAGAGGAGTGAGCA	ChIP
TUB2-R	ACGCATCTTCGGTTGGATGAGTGA	ChIP

Full uncropped blots

Figure 2d

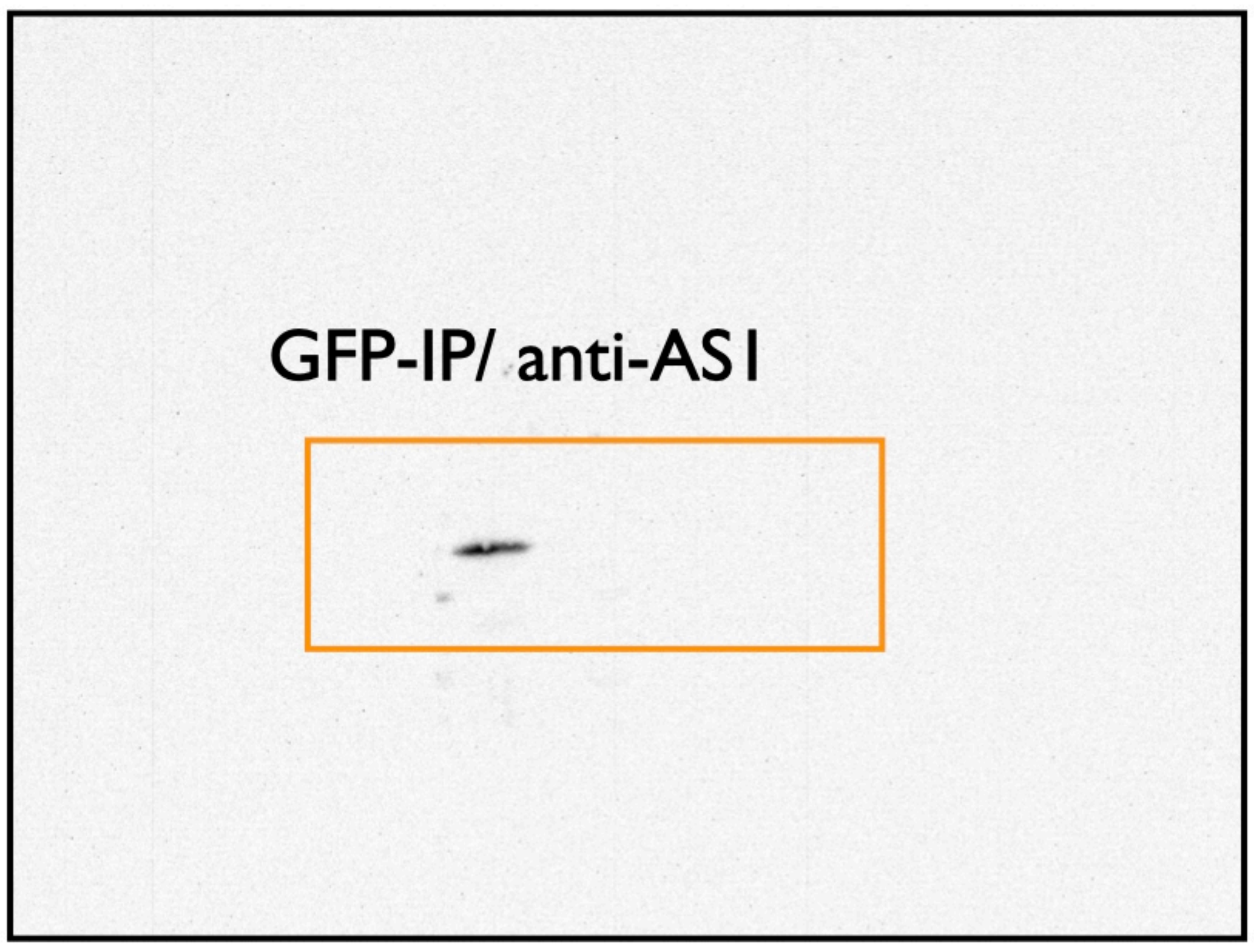

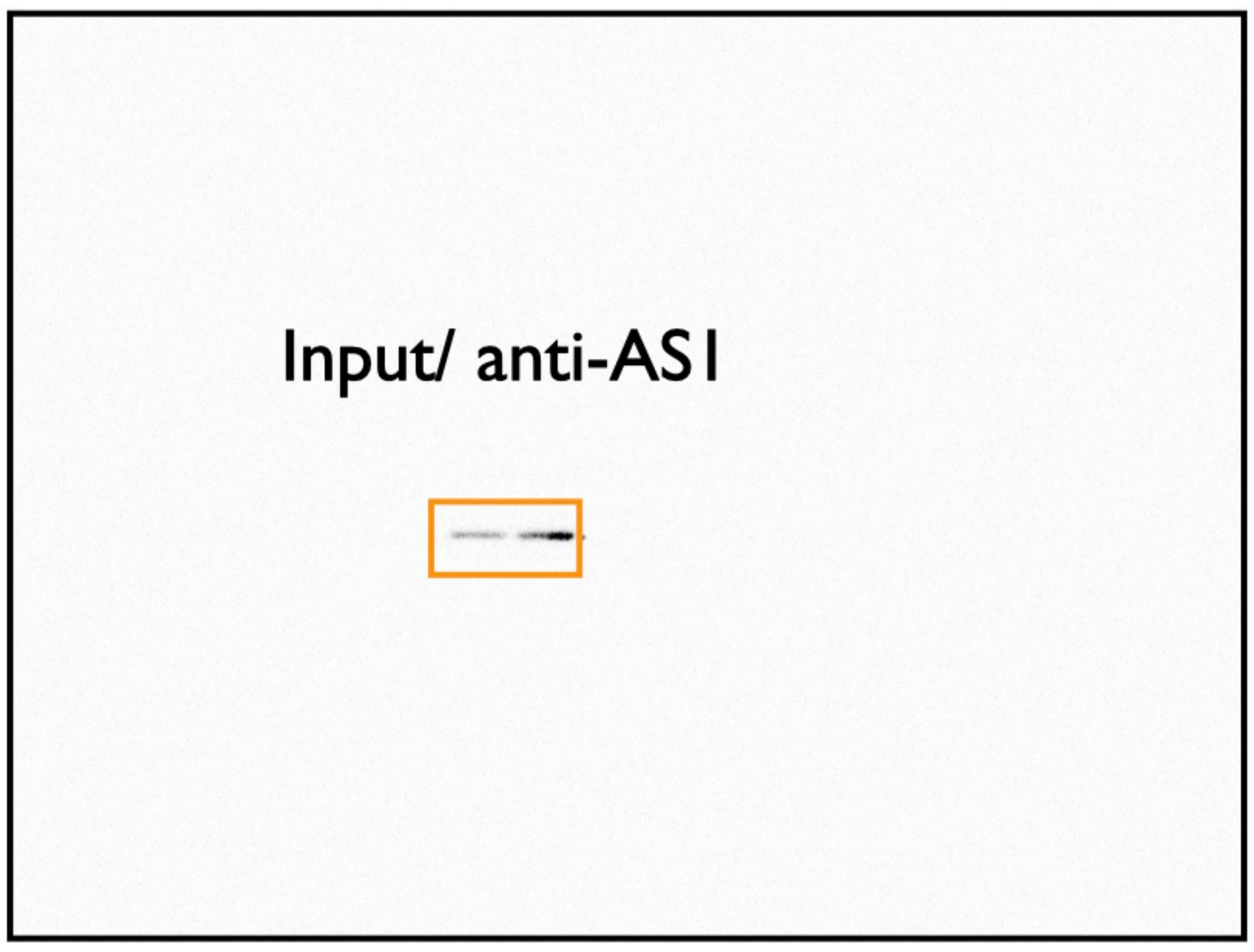

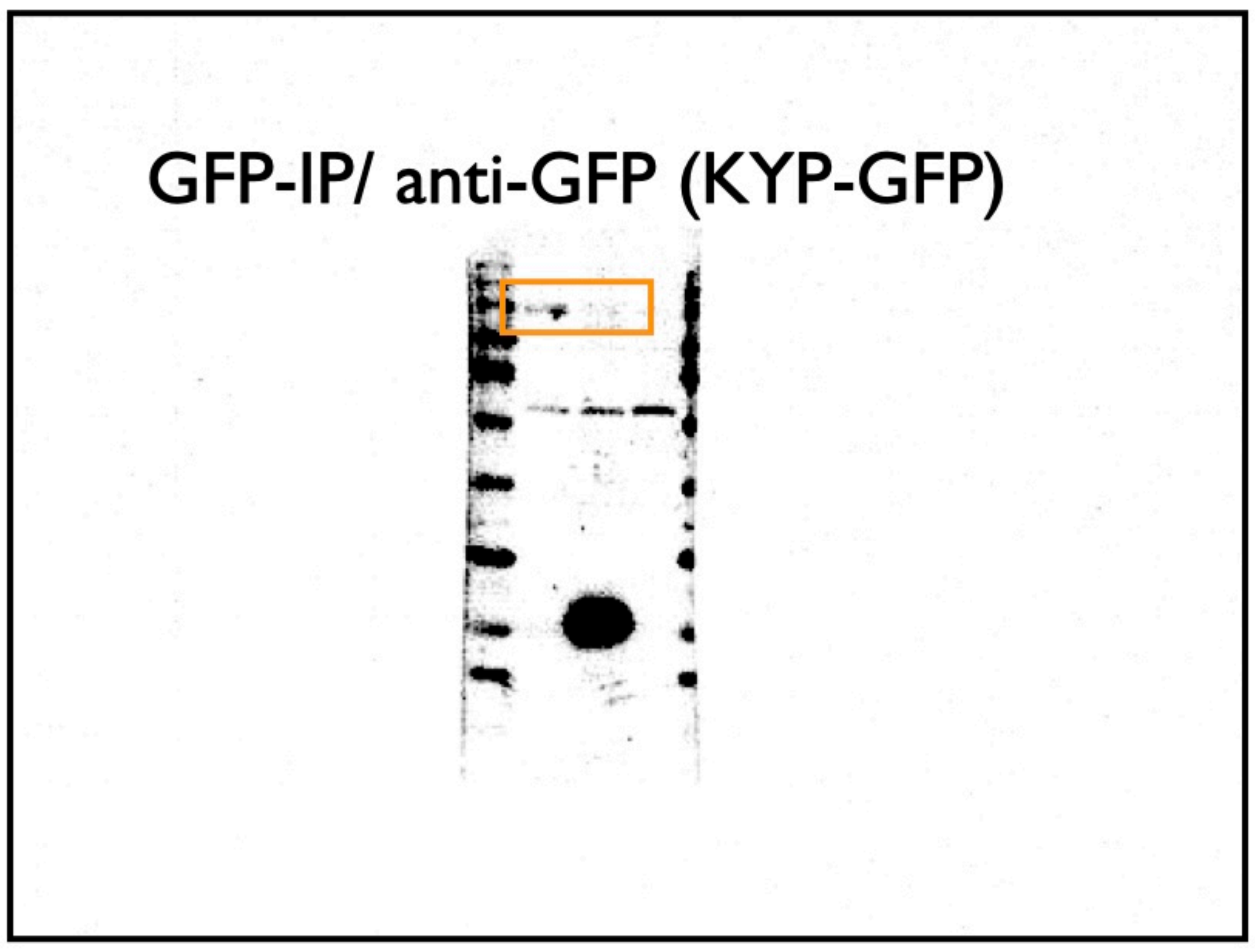

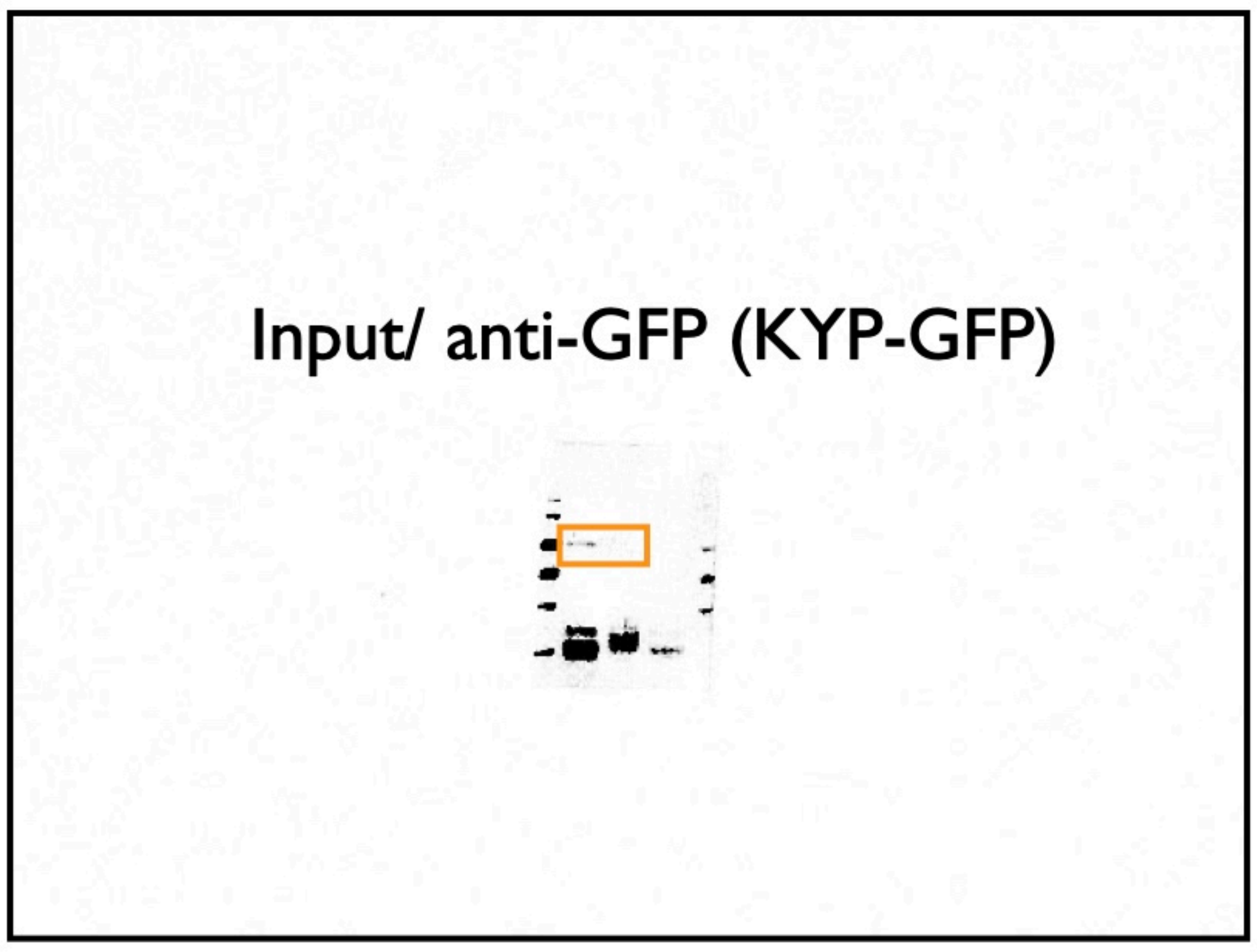

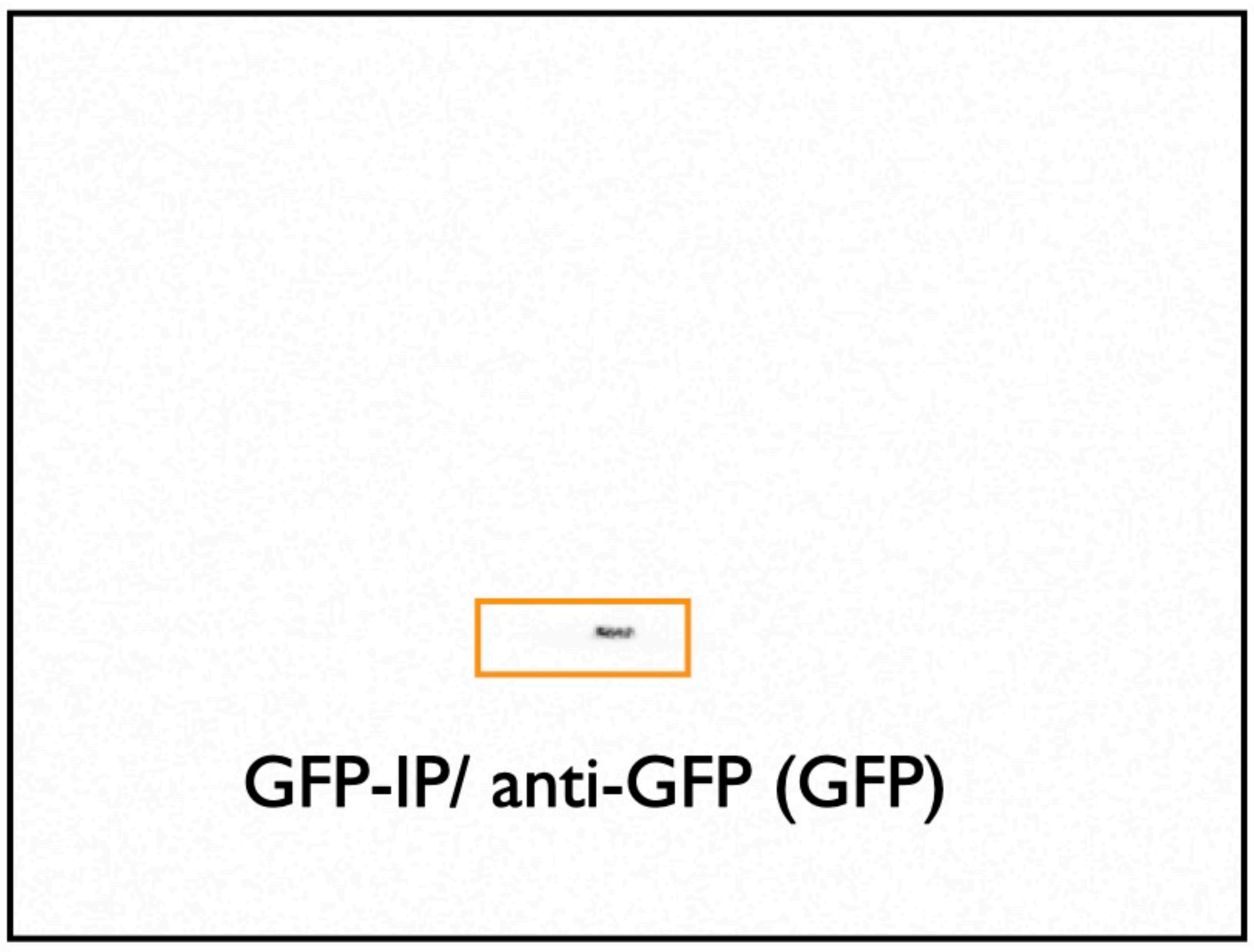

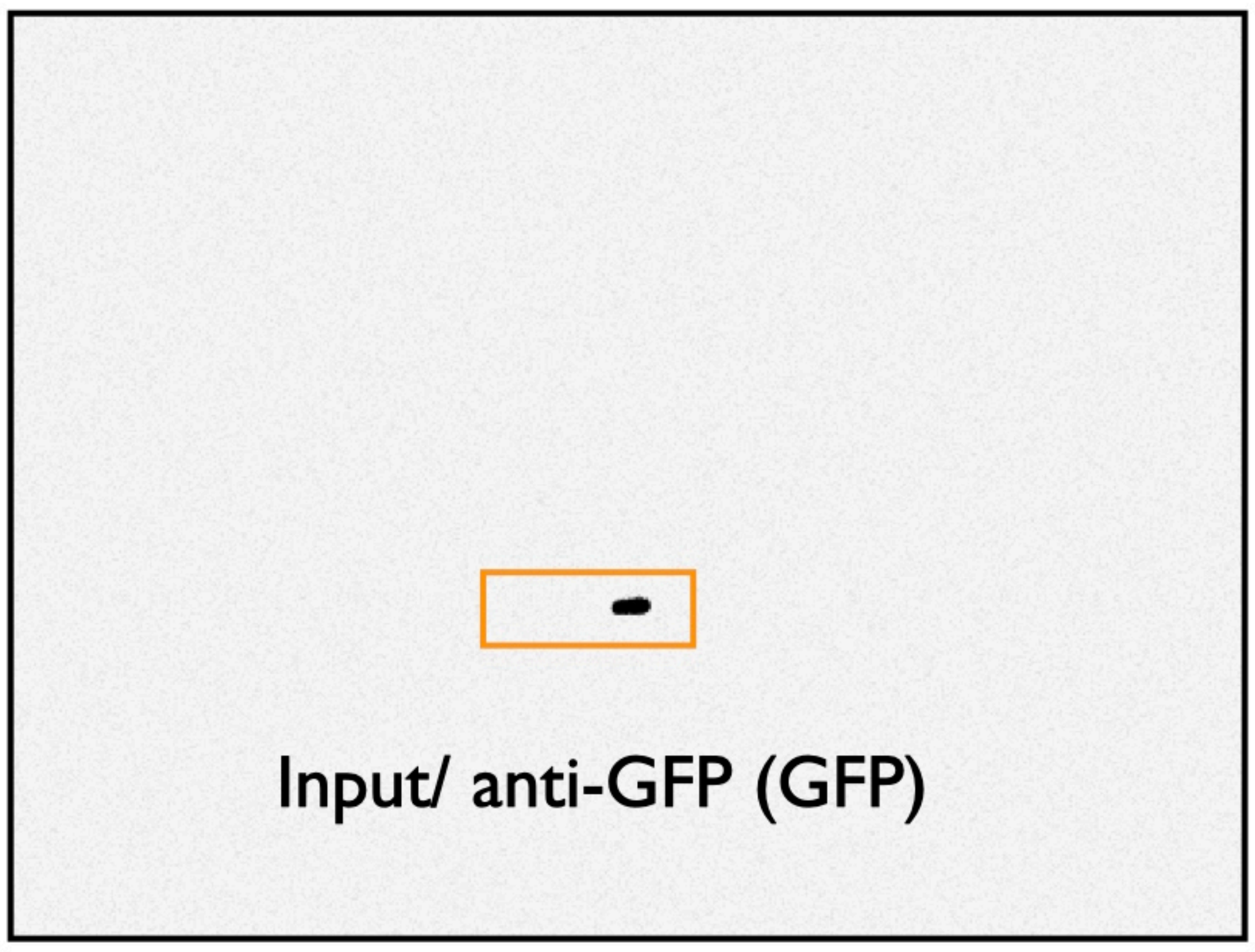

Figure S2c

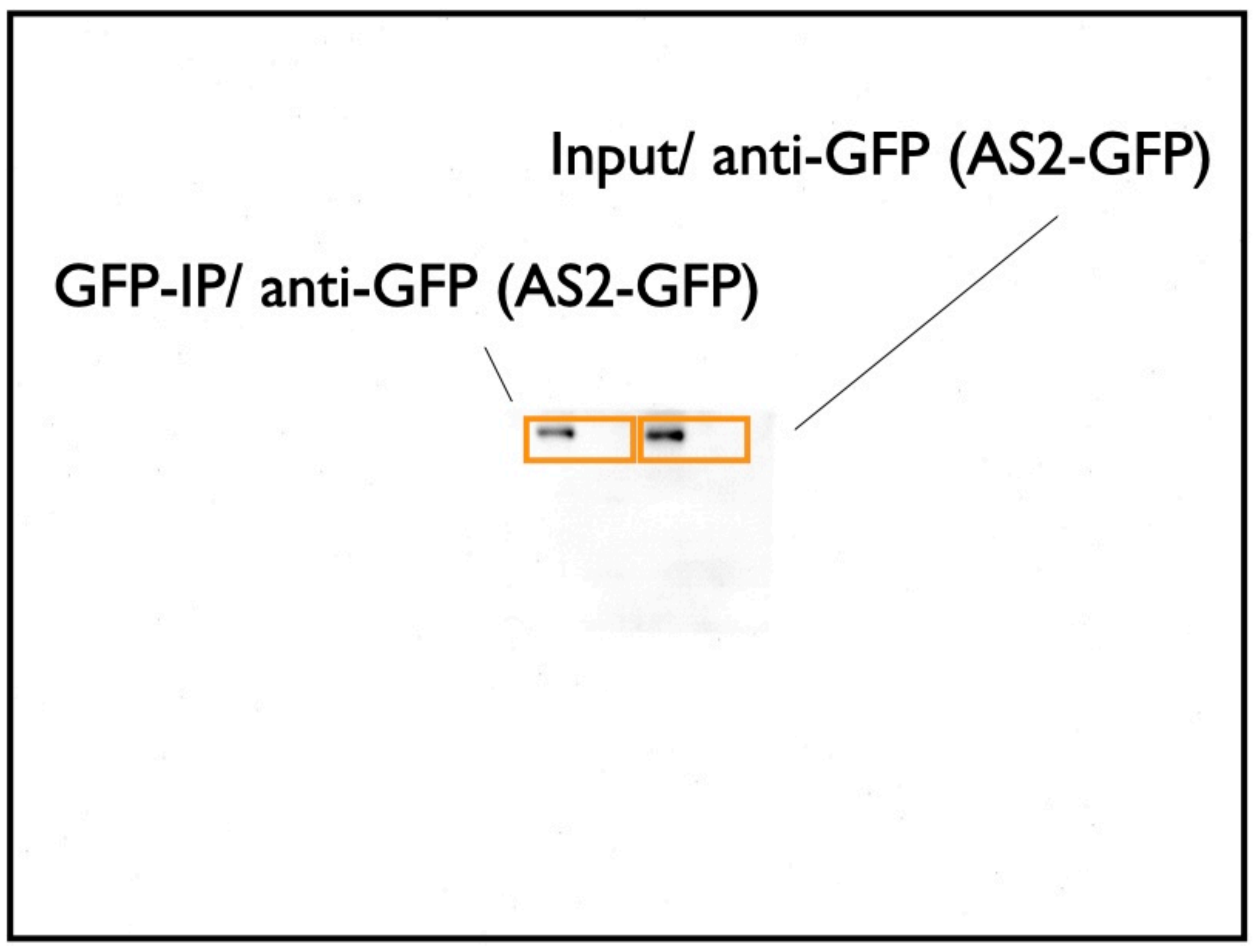

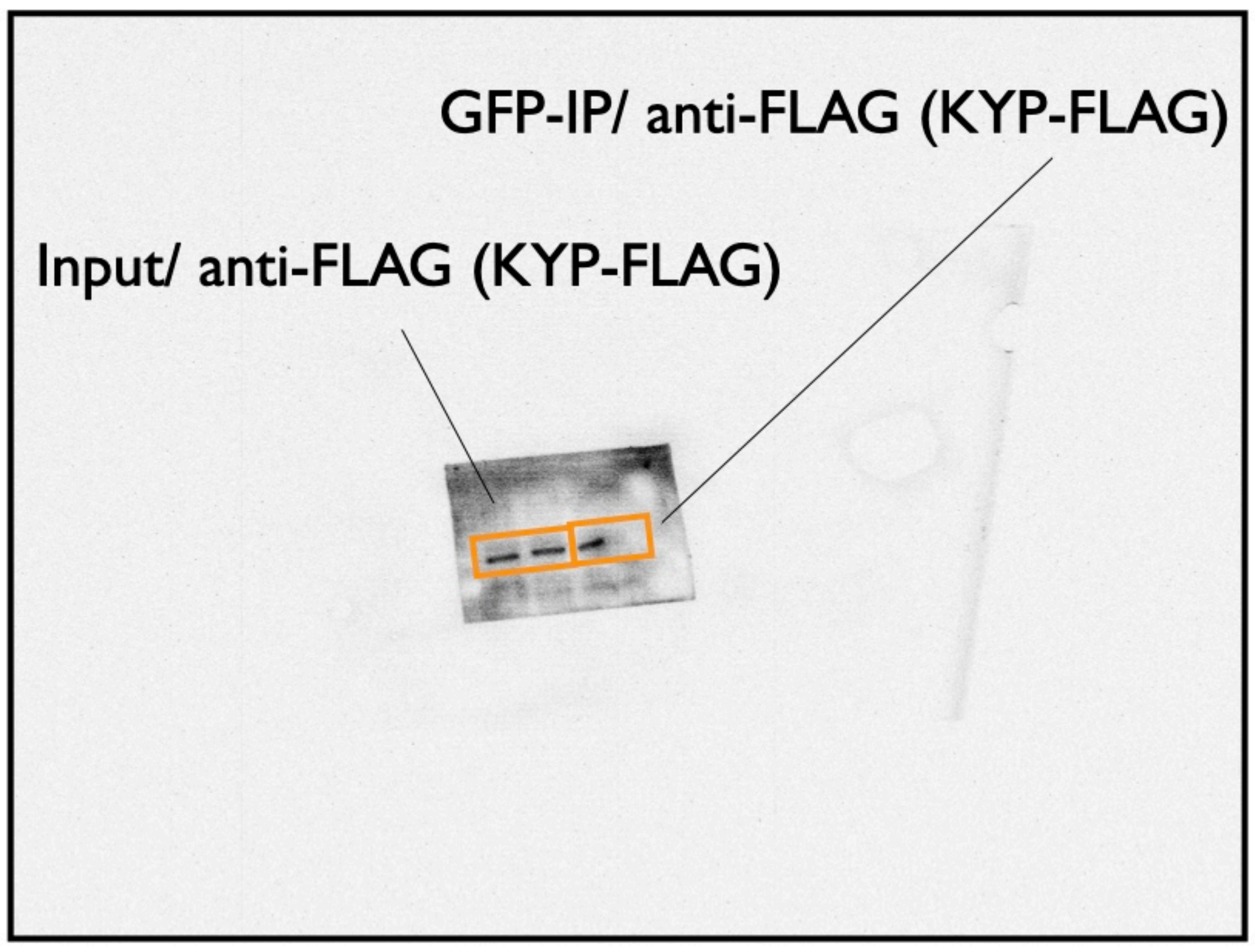

Supplement: Supplementary file 2 — Supplementary Information [file 42003_2023_4607_MOESM2_ESM.pdf]
