# Supplementary material for: Arabidopsis histone H3 lysine 9 methyltransferases KYP/SUVH5/6 are involved in leaf development by interacting with AS1-AS2 to repress KNAT1 and KNAT2
Source: Commun Biol. 2023 Feb 24;6:219. doi: 10.1038/s42003-023-04607-6 (PMC9958104; doi:10.1038/s42003-023-04607-6)
Supplement: Supplementary file 3 — Description of Additional Supplementary Files [file 42003_2023_4607_MOESM3_ESM.pdf]

Description of Additional Supplementary Files

File name: Supplementary Data 1

Description: Genomic regions targeted by KYP.

File name: Supplementary Data 2

Description: The source data behind the graphs in the paper.
