# Supplementary material for: Arabidopsis histone H3 lysine 9 methyltransferases KYP/SUVH5/6 are involved in leaf development by interacting with AS1-AS2 to repress KNAT1 and KNAT2
Source: Commun Biol. 2023 Feb 24;6:219. doi: 10.1038/s42003-023-04607-6 (PMC9958104; doi:10.1038/s42003-023-04607-6)
Supplement: Supplementary file 6 — Reporting Summary [file 42003_2023_4607_MOESM6_ESM.pdf]

nature portfolio

Corresponding author(s): Keqiang Wu

Last updated by author(s): 2022/08-04

# Reporting Summary

Nature Portfolio wishes to improve the reproducibility of the work that we publish. This form provides structure for consistency and transparency in reporting. For further information on Nature Portfolio policies, see our Editorial Policies and the Editorial Policy Checklist.

## Statistics

For all statistical analyses, confirm that the following items are present in the figure legend, table legend, main text, or Methods section.

| n/a | Confirmed | |
|---|---|---|
| ☐ | ☒ | The exact sample size (*n*) for each experimental group/condition, given as a discrete number and unit of measurement |
| ☐ | ☒ | A statement on whether measurements were taken from distinct samples or whether the same sample was measured repeatedly |
| ☐ | ☒ | The statistical test(s) used AND whether they are one- or two-sided<br>*Only common tests should be described solely by name; describe more complex techniques in the Methods section.* |
| ☐ | ☒ | A description of all covariates tested |
| ☐ | ☒ | A description of any assumptions or corrections, such as tests of normality and adjustment for multiple comparisons |
| ☐ | ☒ | A full description of the statistical parameters including central tendency (e.g. means) or other basic estimates (e.g. regression coefficient) AND variation (e.g. standard deviation) or associated estimates of uncertainty (e.g. confidence intervals) |
| ☐ | ☒ | For null hypothesis testing, the test statistic (e.g. *F*, *t*, *r*) with confidence intervals, effect sizes, degrees of freedom and *P* value noted<br>*Give P values as exact values whenever suitable.* |
| ☐ | ☒ | For Bayesian analysis, information on the choice of priors and Markov chain Monte Carlo settings |
| ☐ | ☒ | For hierarchical and complex designs, identification of the appropriate level for tests and full reporting of outcomes |
| ☐ | ☒ | Estimates of effect sizes (e.g. Cohen's *d*, Pearson's *r*), indicating how they were calculated |

*Our web collection on statistics for biologists contains articles on many of the points above.*

## Software and code

Policy information about availability of computer code

| | |
|---|---|
| Data collection | Only public software/codes were used. |
| Data analysis | Only public software/codes were used. |

For manuscripts utilizing custom algorithms or software that are central to the research but not yet described in published literature, software must be made available to editors and reviewers. We strongly encourage code deposition in a community repository (e.g. GitHub). See the Nature Portfolio guidelines for submitting code & software for further information.

## Data

Policy information about availability of data

All manuscripts must include a data availability statement. This statement should provide the following information, where applicable:

- Accession codes, unique identifiers, or web links for publicly available datasets
- A description of any restrictions on data availability
- For clinical datasets or third party data, please ensure that the statement adheres to our policy

----------
To review GEO accession GSE195735:
Go to https://www.ncbi.nlm.nih.gov/geo/query/acc.cgi?acc=GSE195735

Enter token mxilkwmqflqhxej into the box
----------

## Human research participants

Policy information about studies involving human research participants and Sex and Gender in Research.

| | |
|---|---|
| Reporting on sex and gender | *Use the terms sex (biological attribute) and gender (shaped by social and cultural circumstances) carefully in order to avoid confusing both terms. Indicate if findings apply to only one sex or gender; describe whether sex and gender were considered in study design whether sex and/or gender was determined based on self-reporting or assigned and methods used. Provide in the source data disaggregated sex and gender data where this information has been collected, and consent has been obtained for sharing of individual-level data; provide overall numbers in this Reporting Summary. Please state if this information has not been collected. Report sex- and gender-based analyses where performed, justify reasons for lack of sex- and gender-based analysis.* |
| Population characteristics | *Describe the covariate-relevant population characteristics of the human research participants (e.g. age, genotypic information, past and current diagnosis and treatment categories). If you filled out the behavioural & social sciences study design questions and have nothing to add here, write "See above."* |
| Recruitment | *Describe how participants were recruited. Outline any potential self-selection bias or other biases that may be present and how these are likely to impact results.* |
| Ethics oversight | *Identify the organization(s) that approved the study protocol.* |

Note that full information on the approval of the study protocol must also be provided in the manuscript.

# Field-specific reporting

Please select the one below that is the best fit for your research. If you are not sure, read the appropriate sections before making your selection.

☒ Life sciences ☐ Behavioural & social sciences ☐ Ecological, evolutionary & environmental sciences

For a reference copy of the document with all sections, see nature.com/documents/nr-reporting-summary-flat.pdf

# Life sciences study design

All studies must disclose on these points even when the disclosure is negative.

| | |
|---|---|
| Sample size | 20-50 plants of each group |
| Data exclusions | no data exclusion |
| Replication | three success biological replicates |
| Randomization | Use WT plant as control, and compared to mutant plants |
| Blinding | Each sample were random selected. |

# Reporting for specific materials, systems and methods

We require information from authors about some types of materials, experimental systems and methods used in many studies. Here, indicate whether each material, system or method listed is relevant to your study. If you are not sure if a list item applies to your research, read the appropriate section before selecting a response.

## Materials & experimental systems

| n/a | Involved in the study | |
|---|---|---|
| ☐ | ☒ | Antibodies |
| ☒ | ☐ | Eukaryotic cell lines |
| ☒ | ☐ | Palaeontology and archaeology |
| ☒ | ☐ | Animals and other organisms |
| ☒ | ☐ | Clinical data |
| ☒ | ☐ | Dual use research of concern |

## Methods

| n/a | Involved in the study | |
|---|---|---|
| ☐ | ☒ | ChIP-seq |
| ☒ | ☐ | Flow cytometry |
| ☒ | ☐ | MRI-based neuroimaging |

## Antibodies

| | |
|---|---|
| Antibodies used | Anti-GFP (Santa Cruz Biotechnologies, catalog no. SC-9996; 1:3000 dilution) and anti-AS1 (SIGMA catalog no. M2; 1:3000 dilution) antibodies were used as primary antibodies for Western blot. Antibodies against anti-FLAG (SIGMA, catalog no. M2), H3Ac (Millipore, catalog no. 06-599) or H3K9me2 (diagenode, C15410060) were used for ChIP. |
| Validation | Anti-GFP (Santa Cruz Biotechnologies, catalog no. SC-9996) https://www.scbt.com/p/gfp-antibody-b-2?requestFrom=search<br>anti-AS1(Luo et al., 2012) https://doi.org/10.1371/journal.pgen.1003114<br>anti-FLAG (SIGMA, catalog no. M2) https://www.sigmaaldrich.com/TW/en/product/sigma/f3165<br>H3Ac (Millipore, catalog no. 06-599) https://www.merckmillipore.com/JP/ja/product/Anti-acetyl-Histone-H3-Antibody,MM_NF-06-599<br>H3K9me2 (diagenode, C15410060) https://www.diagenode.com/en/p/h3k9me2-polyclonal-antibody-classic-50-ug-44-ul |

## ChIP-seq

### Data deposition

☒ Confirm that both raw and final processed data have been deposited in a public database such as GEO.

☒ Confirm that you have deposited or provided access to graph files (e.g. BED files) for the called peaks.

| | |
|---|---|
| Data access links<br>*May remain private before publication.* | ----------<br>To review GEO accession GSE195735:<br>Go to https://www.ncbi.nlm.nih.gov/geo/query/acc.cgi?acc=GSE195735<br>Enter token mxilkwmqflqhxej into the box<br>---------- |
| Files in database submission | GSM5849480 KYP:3xFLAG-IP-1<br>GSM5849481 KYP:3xFLAG-IP-2<br>GSM5849482 KYP:3xFLAG-input |
| Genome browser session<br>(e.g. UCSC) | ----------<br>To review GEO accession GSE195735:<br>Go to https://www.ncbi.nlm.nih.gov/geo/query/acc.cgi?acc=GSE195735<br>Enter token mxilkwmqflqhxej into the box<br>---------- |

### Methodology

| | |
|---|---|
| Replicates | Two independent KYPpro::KYP:3xFLAG/kyp transgenic lines were used as biological replicates for ChIP-seq experiment. |
| Sequencing depth | Approximately 24 and 16 million mapped reads of KYPpro::KYP:3xFLAG transgenic line #1 and #4 were used for analysis (pair-end, 150bp). |
| Antibodies | anti-FLAG (SIGMA, catalog no. M2) https://www.sigmaaldrich.com/TW/en/product/sigma/f3165 |
| Peak calling parameters | MACS2 were used for peak calling with default setting. |
| Data quality | 3,924 genomic regions targeted by KYP were identified by MACS2. |
| Software | The alignments were first converted to Wiggle (WIG) files using deepTools. The data were then imported into the Integrated Genome Viewer (IGV) (Thorvaldsdottir et al., 2013) for visualization. The distribution of the ChIP binding peaks was analyzed with ChIPseeker (Table S2) (Yu et al., 2015), and a high-read random Arabidopsis genomic region subset (1,350,000 regions) was used to represent the ratio of the total Arabidopsis genomic regions. To identify DNA motifs enriched sites, 400-bp sequences encompassing each peak summit (200 bp upstream and 200 bp downstream) were extracted and searched for enriched DNA motifs using MEME-ChIP with the default parameters (Machanick and Bailey, 2011). |
